# Evaluation of the effect of direct-acting antiviral agents on melatonin level and lipid peroxidation in chronic hepatitis C patients

**DOI:** 10.3389/fphar.2023.1128016

**Published:** 2023-08-08

**Authors:** Nageh Ahmed El-Mahdy, Sabry Abou-Saif, Medhat Ismail Abd EL hamid, Heba M. Hashem, Mohamed Anwar Hammad, Sally El-Sayed Abu-Risha

**Affiliations:** ^1^ Department of Pharmacology and Toxicology, Faculty of Pharmacy, Tanta University, Tanta, Egypt; ^2^ Department of Tropical Medicine and Infectious Diseases, Faculty of Medicine, Tanta University, Tanta, Egypt; ^3^ Department of Pharmacology, Faculty of Medicine, Al-Azhar University, Cairo, Egypt; ^4^ Department of Pharmacy Practice, Faculty of Pharmacy, Sinai University, El-Arish, Egypt; ^5^ Department of Clinical Pharmacy, Faculty of Clinical Pharmacy, Al-Baha University, Al-Baha, Saudi Arabia

**Keywords:** melatonin, malondialdehyde, direct-acting agents (DAAs), hepatitis C virus (HCV), oxidatie stress

## Abstract

**Background:** Oxidative stress and its end products, such as malondialdehyde (MDA) play a leading role in the pathogenesis of hepatitis C. Melatonin is a hormone that helps regulate circadian rhythms, which likely play a role in infectious diseases in terms of susceptibility, clinical expression, and outcome.

**Objective:** The present study was conducted to assess serum malondialdehyde and melatonin levels in patients with chronic hepatitis C infection before and after the intake of direct-acting antivirals.

**Method:** Forty hepatitis C patients were the subjects of this study. While ten healthy volunteers who matched in age and socioeconomic status served as the control subjects. Malondialdehyde and melatonin were assayed in the serum of the three groups, and the results were statistically analyzed.

**Results:** Hepatitis C patients had significantly higher malondialdehyde (*p* < 0.001) but significantly lower melatonin (*p* < 0.001) as compared to the healthy controls. After 12 weeks of treatment with direct-acting antivirals, the malondialdehyde level decreased significantly (*p* < 0.001) and the melatonin level increased significantly (*p* < 0.001). A significant negative correlation between malondialdehyde and melatonin was observed.

**Conclusion**: The present findings suggest that treatment of hepatitis C patients with Direct-acting antivirals improves liver function parameters and antioxidant profiles.

## Introduction

The hepatitis C virus (HCV) is a serious public health issue worldwide. Egypt has the highest global prevalence of HCV (Waked, 2022). 400,000 individuals worldwide are predicted to die from cirrhosis and hepatocellular carcinoma each year as a result of HCV, which affects an estimated 71 million people globally (HCC) (Moon et al., 2020). Despite the fact that HCV can now be accurately detected and treated with DAAs, which has cure rates of greater than 95% in 8–12 weeks, one of the most astonishing medical advancements in recent memory, viral hepatitis mortality increased by 22% between 2000 and 2015 (Schwander et al., 2022).

HCV replicates in the cytoplasm and causes chronic infections that may eventually result in cirrhosis, chronic hepatitis, and hepatocellular carcinoma (HCC) (Vrazas et al., 2022). It has been discovered that oxidative stress plays a significant role in HCV genome translation, which is mediated via PERK-mediated suppression of cap-dependent translation (Ivanov et al., 2013). Researchers previously discovered that the likely mechanism for viral escape from the immune system is reactive oxygen species (ROS)-generated viral genomic heterogeneity (Paracha et al., 2013).

Oxidative stress results in damage and the possibility of cell death due to the oxidation of many cellular components such as DNA, proteins, and lipids (lipid peroxidation) (apoptosis) (Avery, 2011). Once this process is started, cellular damage and the release of pro-inflammatory cytokines cycle continuously, causing hepatic inflammation, fibrosis, and cirrhosis (Vuppalanchi et al., 2011).

It has long been believed that one of the main effects of rogue free radicals is the peroxidation of membrane lipids. Polyunsaturated fatty acids undergo a chemical change as a result of lipid peroxidation, which also leads to the breakdown of the structural integrity of cellular and subcellular membranes. The dynamic properties of the lipid bilayer play a crucial role in controlling numerous of important physiological activities in the cell. Consequently, the disturbance of structural features brought on by oxidative stress has negative effects on cellular function (García et al., 2014).

Unsaturated reactive aldehydes and malondialdehyde (MDA) are two examples of lipid peroxidation end products that have been utilized as indicators of oxidative stress. As an indirect indicator of oxidative stress, MDA is relatively stable and simple to quantify in serum, plasma and urine (Kartavenka et al., 2022).

N-acetyl-5-methoxytryptamine, often known as melatonin, is a widely disseminated chemical in nature with a wide range of uses. To protect the morphological and functional components of the cell membrane from damage by free radicals, melatonin demonstrates exceptional functional properties (Radogna et al., 2010).

Melatonin, usually referred to as the “sleep hormone,” has numerous important features, including being anti-inflammatory and anti-apoptotic (Tarocco et al., 2019).

The hypothalamic suprachiasmatic nucleus controls circadian rhythms and clock gene expression, and the pineal gland produces melatonin. Although the liver produces melatonin and has its own separate circadian rhythms and expressions, the brain senses light through the retinas and controls rhythms and melatonin secretion throughout the body. Circadian rhythm disturbance or clock gene expression may encourage the development of liver steatosis, inflammation, or cancer, according to earlier studies linking several liver disorders and circadian rhythms. The powerful antioxidant benefits of melatonin are well known. ROS and oxidative stress are produced in the liver by excessive fatty acid accumulation or alcohol consumption, which can harm the liver (Sato et al., 2020).

HCV infection, which results in continuous liver inflammation, can develop chronic hepatitis, cirrhosis, and HCC. Interferon (IFN)-based regimens, which have a low cure rate and the potential for serious side effects, were the only anti-HCV treatments available in the past. However, a number of oral anti-HCV medications (direct-acting antivirals, or DAAs) have become available recently (Endo et al., 2017). DAAs were first used with ribavirin and PEGIFN to boost response rates, but this increased toxicity (Pawlotsky, 2014). The development of interferon-free regimens with much improved therapeutic tolerance has been made possible by the very effective combination of DAAs that target several stages of the viral life cycle. The majority of patient populations’ cure rates have now reached 90% thanks to well-tolerated oral regimens (Feld et al., 2014).

The National Committee for Control of Viral Hepatitis in Egypt states that patients fall into one of the two categories below: A) Easy-to-treat group meeting the following requirements: treatment-naive, total serum bilirubin of 1.2 mg/dL or lower, serum albumin of 3.5 g/dL or higher, INR of 1.2 or lower, and platelet count of 150,000/mm3 or higher. This group is eligible to receive treatment for a total of 12 weeks with any of the following regimens: sofosbuvir and daclatasvir or Qurevo^®^ (Ombitasvir, Paritaprevir, and Ritonavir), as well as ribavirin. B) Group that is difficult to treat according to these standards: Having received Peg-INF treatment, having total serum bilirubin levels greater than 1.2 mg/dL, serum albumin levels lower than 3.5 g/dL, having an INR greater than 1.2, and having a platelet count below 150,000/mm3. This group is eligible to get a 12-week course of sofosbuvir, daclatasvir, and ribavirin treatment (Allam et al., 2022).

Sofosbuvir, daclatasvir with or without ribavirin, have been exclusively used for all patients in the Egyptian national program for managing HCV since early 2016 due to the high response rates with the locally produced generics, which support the use of low-cost generics in similar programs in limited resource settings (Waked, 2022).

## Materials and methods

### Study design

This cohort study was performed on 10 healthy individuals (6 men, 4 women) who served as controls, or Group I, and 40 Egyptian patients who had chronic infection with hepatitis C treated with a combination of IFN-free DAAs at the Hepatology and Virology outpatient clinic at Tanta University Hospital during the period from May 2022 to July 2022 after the study was approved by the Institutional Review Board of Tanta University (IRB study protocol code: TP/RE/9/4/22 ph-001). The study was registered as a clinical trial (ClinicalTrials.gov identifier: NCT05372874). Written informed consent was obtained from all participants. HCV Patients were divided into two groups (Group II and Group III). Group II included 20 patients with CHC infection who were treated with DAAs (one sofosbuvir 400 mg tablet and one daclatasvir 60 mg capsule once a day) for 12 weeks. Patients in this group had one or more of the following conditions: treatment naïve, total serum bilirubin of 1.2 mg/dL or lower, serum albumin of 3.5 g/dL or higher, INR of 1.2 or lower, and a platelet count of 150,000/mm^3^ or higher.

**FIGURE 1:**
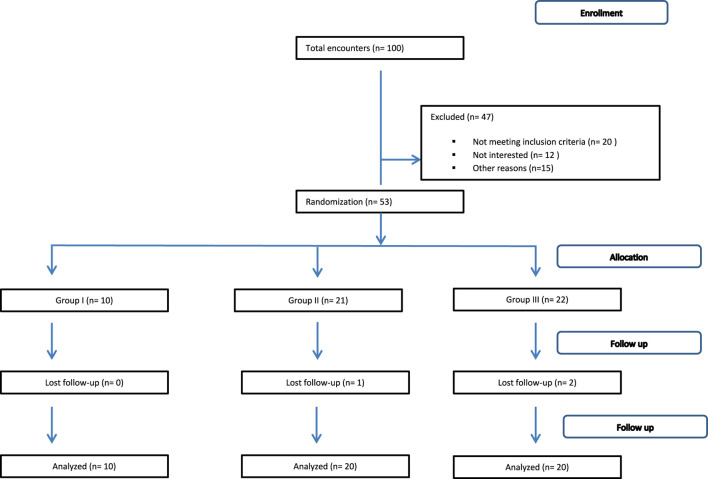
Flowchart illustrating the participants’ screening, enrollment, and randomization.

Group III included 20 patients with CHC infection who were treated with DAAs (one sofosbuvir 400 mg tablet, one daclatasvir 60 mg capsule once a day, and 600–1000 ribavirin) according to tolerance for 12 weeks (this group belongs to the difficult-to-treat group according to the National Committee for Control of Viral Hepatitis in Egypt, 2016). Patients in this group had one or more of the following conditions: total serum bilirubin is higher than 1.2 mg/dL, serum albumin is lower than 3.5 g/dL, INR is higher than 1.2, and platelet count is less than 150,000/mm^3^. Figure 1.

The enrolled patients, besides being HCV positive, were recruited based on specific eligibility criteria, including the absence of HBV infection, renal insufficiency, Hepatocellular carcinoma (HCC) or other types of malignancy, current use of melatonin or any medications that have interactions with melatonin, consuming a lot of caffeine, being heavy smokers, or working night shifts. Also, patients were excluded for another reasons, such as pregnancy or lactation, alcohol consumption, liver transplantation, the presence of neurological disorders or major psychiatric diseases, patients with cerebral disease or trauma that may affect the pineal gland, and difficulty of follow-up. The control group comprised 10 healthy volunteers who met the same eligibility criteria, i.e., they were HCV-negative and were age- and gender-matched.

Patients were followed-up by weekly phone calls and directed to meet at monthly intervals to assess their average sleep time, adherence to the study medications, and any medication adverse effects.

### Clinical and laboratory assessment

All participants were subjected to a full history and a thorough clinical examination, with a focus on sex, age, and route of HCV transmission. Two fasting blood samples, 10 mL each, were withdrawn from every hepatitis C patient at 9 a.m.: one before the recommended antiviral therapy intake and the second after 12 weeks of treatment. Complete blood cell counts were done immediately. Then, the remaining blood was allowed to clot at room temperature and centrifuged at 3,000 rpm for 10 min. Sera were separated. 2 mL of serum from each sample was kept frozen at −70°C until the quantitative determination of MDA and melatonin levels. The remaining serum of each sample was used immediately for the determination of liver function parameters such as alanine aminotransferase (ALT), aspartate aminotransferase (AST), total and direct bilirubin, Gamma-glutamyl transferase (GGT), albumin and international normalized ratio (INR). Fasting lipid profile [total cholesterol, high-density lipoprotein (HDL), and low-density lipoprotein (LDL)] and serum creatinine were also determined.

#### Analysis of MDA

Lipid peroxidation was estimated by determination of thiobarbituric acid-reactive substance (TBARS) content, reflective of the MDA end-product of membrane oxidation, using a commercial kit (Biodiagnostic, Giza, Egypt, Catalog No. MD 25029).

#### Analysis of melatonin

Colorimetric melatonin levels were measured by a double antibody sandwich ELISA using commercially available ELISA kits and in accordance with the manufacturer’s instructions (SunRed; SunRed Biological Technology Co. Ltd., Shanghai, China, Catalog No. 201-12-1014). At the same time, 10 healthy subjects were similarly investigated.

#### Assessment of HCV-RNA

Assessment of HCV-RNA load at baseline and at the end of treatment (12 weeks). Undetectable HCV-RNA at the end of treatment response was defined as undetectable HCV-RNA at the completion of treatment.

#### Data analysis

The data were analyzed using a statistical package for the social science (SPSS) version 20.0 software (SPSS Inc., Chicago, IL, United States). Quantitative data were expressed as mean ± Standard deviation. Qualitative data are expressed as numbers and percentages and analyzed by Chi–square test (X2). Student’s t-test was used to compare means between the two groups. Multiple comparisons were performed by one-way analysis of variance (ANOVA) followed by the Chi-square test for multiple comparisons. Correlations were analyzed using the Pearson test. *p*-values of 0.05 were considered statistically significant.

## Results

Our cohort was conducted on 50 individuals divided into 3 groups: group I: 10 patients as a control group, group II: 20 patients with CHC infection who were treated with DAAs (sofosbuvir/daclatasvir) for 12 weeks; and group III: 20 patients with CHC infection who were treated with DAAs (sofosbuvir/daclatasvir/ribavirin) for 12 weeks. Table 1 shows that no significant differences were observed in age or gender between groups (*p* > 0.05).

**TABLE 1:** Demographic data of the study participants.

Parameter	Group I: (*n* = 10)	Group II: (*n* = 20)	Group III: (*n* = 20)	*p*-value
Age^a^, y	48.300 ± 4.322	48.900 ± 8.416	55.250 ± 15.986	0.160
Mean ± SD				
Sex^a^				0.353
Male	6 (60.00 %)	7 (35.00 %)	7 (35.00 %)	
Female	4 (40.00 %)	13 (65.00 %)	13 (65.00 %)	

*p* < 0.05 is considered significant.

Values are presented as mean (SD).

^a^ Values are presented as n (%).

Table 2 shows that CHC patients (pre-treatment, group II and group III) had significantly higher ALT, AST, GGT, and MDA than the control group (group I) (*p* < 0.001, *p* = 0.009, *p* < 0.001, *p* < 0.001, respectively), but significantly lower albumin, hemoglobin, cholesterol, LDL, HDL, and melatonin (*p* < 0.001, *p* = 0.001, *p* < 0.001, *p* = 0.004, *p* = 0.014, *p* < 0.001, *p* < 0.001 respectively). However, there were insignificant differences between CHC patients (pre-treatment, group II and group III) and the control group (group I) regarding serum creatinine level (*p* = 0.077). Also, group II had significantly higher total bilirubin, direct bilirubin, INR, prothrombin time, and WBCs than the control group (group I) (*p* = 0006, *p* = 0.008, *p* = 0.008, *p* = 0.031, *p* < 0.001 respectively), but significantly lower Platelets (*p* < 0.001) than the control group (group I).

**TABLE 2:** Baseline selected laboratory data of all patients in the 3 study group.

Parameter	Group I: (*n* = 10)	Group II: (*n* = 20)	Group III: (*n* = 20)	*p*-value (among all groups)
ALT, IU/L	23.100 ± 2.470	38.700 ± 4.067^$^	91.685 ± 44.368^$#^	<0.001*
AST, IU/L	25.900 ± 1.729	40.450 ± 3.649^$^	53.235 ± 34.641^$^	0.009*
BIL-T, mg/dL	0.853 ± 0.049	0.902 ± 0.099	1.024 ± 0.204^$#^	0.006*
BIL-D, mg/dL	0.120 ± 0.027	0.143 ± 0.017	0.202 ± 0.111^$#^	0.008*
Albumin, g/dL	4.450 ± 0.158	3.940 ± 0.185^$^	3.790 ± 0.484^$^	<0.001*
INR, IU	1.079 ± 0.152	1.094 ± 0.067	1.200 ± 0.138^$#^	0.008*
PT, sec	13.000 ± 1.826	13.500 ± 1.680	14.612 ± 1.626^$^	0.031*
Hb, g/dL	14.250 ± 1.461	11.818 ± 1.489^$^	12.268 ± 1.562^$^	0.001*
Platelets, 10^9^/L	238.800 ± 60.487	207.650 ± 59.063	129.050 ± 43.686^$#^	<0.001*
WBCs, 10^9^/L	5.038 ± 0.698	5.536 ± 1.412	8.549 ± 2.140^$#^	<0.001*
Creatinine, mg/dL	0.838 ± 0.116	0.875 ± 0.096	0.958 ± 0.196	0.077
GGT, U/L	24.500 ± 1.581	39.000 ± 2.714^$^	42.100 ± 6.512^$^	<0.001*
Cholesterol, mg/dL	179.300 ± 26.277	165.600 ± 7.074^$^	161.950 ± 5.356^$^	0.004*
LDL, mg/dL	129.340 ± 8.935	115.500 ± 18.392^$^	114.835 ± 7.251^$^	0.014*
HDL, mg/dL	58.200 ± 2.201	39.600 ± 2.186^$^	41.000 ± 2.884^$^	<0.001*
MDA, nmol/mL	1.400 ± 0.302	4.270 ± 0.254^$^	4.435 ± 0.292^$^	<0.001*
Melatonin, pg/mL	45.167 ± 3.410	17.970 ± 2.116^$^	17.895 ± 1.927^$^	<0.001*
HCV RNA (IU/mL)	Not detected	1114033.650 ± 1451732.743	955572.300 ± 4176787.175	0.874

*p* < 0.05 indicates a significant difference compared to the healthy normal control group, and # *p* < 0.05 indicates a significant difference compared to group II. ALT, alanine aminotranferase; AST, aspartate transaminase; BIL-T, total bilirubin; BIL-D, direct bilirubin; INR, international normalized ratio; PT, prothrombin time; Hb, hemoglobin; WBCs, white blood cells; GGT, gamma-glutamyl transferase; LDL, low-density lipoprotein; HDL, high-density lipoprotein and MDA, malondialdehyde. *Values are presented as mean (SD). * Significant at *p* < 0.05.

Table 3 shows that after treatment of CHC patients (group II and group III) there were no significant differences compared to the healthy control group (group I) regarding ALT, AST, total bilirubin, direct bilirubin, INR, prothrombin time, serum creatinine level (*p* = 0.612, *p* = 0.873, *p* = 0.651, *p* = 0.069, *p* = 0.536, *p* = 0.346, respectively), while there were significant differences regarding albumin, hemoglobin, cholesterol, LDL, HDL, MDA, and melatonin (*p* < 0.001, *p* =0.004, *p* < 0.001, *p* = 0.026, *p* < 0.001, *p* < 0.001, *p* < 0.001, respectively). However, group III had significant differences regarding platelets, WBCs, and GGT (*p*=<0.001, *p* < 0.001, *p* = 0.001, respectively).

**TABLE 3:** Comparison among the 3 study groups 12 weeks after treatment.

Parameter	Group I: (*n* = 10)	Group II: (*n* = 20)	Group III: (*n* = 20)	*p*-value) among all groups)
ALT, IU/L	23.100 ± 2.470	24.778 ± 7.395	25.050 ± 3.137	0.612
AST, IU/L	25.900 ± 1.729	26.316 ± 6.371	26.800 ± 3.302	0.873
BIL-T, mg/dL	0.853 ± 0.049	0.862 ± 0.129	0.894 ± 0.161	0.651
BIL-D, mg/dL	0.120 ± 0.027	0.138 ± 0.026	0.139 ± 0.011	0.069
Albumin, g/dL	4.450 ± 0.158	4.215 ± 0.201^$^	4.115 ± 0.225^$^	<0.001*
INR, IU	1.079 ± 0.152	1.074 ± 0.053	1.105 ± 0.081	0.536
PT, sec	13.000 ± 1.826	13.125 ± 0.599	13.480 ± 0.574	0.346
Hb, g/dL	14.250 ± 1.461	12.671 ± 1.533^$^	12.499 ± 1.098^$^	0.004*
Platelets, 10^9^/L	238.800 ± 60.487	232.600 ± 50.543	174.950 ± 38.465^$#^	<0.001*
WBCs, 10^9^/L	5.038 ± 0.698	5.124 ± 0.856	7.203 ± 1.436^$#^	<0.001*
Creatinine, mg/dL	0.838 ± 0.116	0.850 ± 0.170	0.938 ± 0.145	2.251
GGT, U/L	24.500 ± 1.581	29.000 ± 7.980	32.750 ± 2.511^$^	0.001*
Cholesterol, mg/dL	179.300 ± 26.277	207.300 ± 14.012^$^	210.450 ± 10.782^$^	<0.001*
LDL, mg/dL	129.340 ± 8.935	141.600 ± 11.686^$^	140.920 ± 13.539^$^	0.026*
HDL, mg/dL	58.200 ± 2.201	51.150 ± 4.648^$^	44.650 ± 3.117^$#^	<0.001*
MDA, nmol/mL	1.400 ± 0.302	3.295 ± 0.437^$^	2.555 ± 0.307^$#^	<0.001*
Melatonin, pg/mL	45.167 ± 3.410	28.273 ± 5.436^$^	43.890 ± 4.106^#^	<0.001*
HCV RNA (IU/mL)	Not detected	Not detected	Not detected	—

*p* < 0.05 indicates a significant difference compared to the healthy normal control group, and # *p* < 0.05 indicates a significant difference compared to group II. ALT, alanine aminotranferase; AST, aspartate transaminase; BIL-T, total bilirubin; BIL-D, direct bilirubin; INR, international normalized ratio; PT, prothrombin time; Hb, hemoglobin; WBCs, white blood cells; GGT, gamma-glutamyl transferase; HDL, high-density lipoprotein; LDL, low-density lipoprotein and MDA, malondialdehyde. *Values are presented as mean (SD). * Significant at *p* < 0.05.

Table 4 shows that there was a significant decrease in ALT, AST, GGT, and MDA (*p* < 0.001, *p* < 0.001, *p* < 0.001, and *p* < 0.001 respectively). However, there was a significant increase in Albumin, hemoglobin, platelets, cholesterol, LDL, HDL, and melatonin (*p* < 0.001, *p* = 0.016, *p* < 0.001, *p* < 0.001, *p* < 0.001, *p* < 0.001, *p* < 0.001) In addition, there were insignificant decreases in total bilirubin, direct bilirubin, INR, prothrombin time, WBCs, and serum creatinine level (*p* = 0.138, *p* = 0.459, *p* = 0.202, *p* = 0.223, *p* = 0.064, *p* = 0.543 respectively).

**TABLE 4:** Laboratories characterization of HCV-patients treated with dual therapy (Group II).

Parameter	Baseline	After 12 weeks of treatment	*p*-value
ALT, IU/L	38.700 ± 4.067	24.778 ± 7.395	<0.001*
AST, IU/L	40.450 ± 3.649	26.316 ± 6.371	<0.001*
BIL-T, mg/dL	0.902 ± 0.099	0.862 ± 0.129	0.138
BIL-D, mg/dL	0.143 ± 0.017	0.138 ± 0.026	0.459
Albumin, g/dL	3.940 ± 0.185	4.215 ± 0.201	<0.001*
INR, IU	1.094 ± 0.067	1.074 ± 0.053	0.202
PT, sec	13.500 ± 1.680	13.125 ± 0.599	0.223
Hb, g/dL	11.818 ± 1.489	12.671 ± 1.533	0.016*
Platelets, 10^9^/L	207.650 ± 59.063	232.600 ± 50.543	<0.001*
WBCs, 10^9^/L	5.536 ± 1.412	5.124 ± 0.856	0.064
Creatinine, mg/dL	0.875 ± 0.096	0.850 ± 0.170	0.543
GGT, U/L	39.000 ± 2.714	29.000 ± 7.980	<0.001*
Cholesterol, mg/dL	165.600 ± 7.074	207.300 ± 14.012	<0.001*
LDL, mg/dL	115.500 ± 18.392	141.600 ± 11.686	<0.001*
HDL, mg/dL	39.600 ± 2.186	51.150 ± 4.648	<0.001*
MDA, nmol/mL	4.270 ± 0.254	3.295 ± 0.437	<0.001*
Melatonin, pg/mL	17.970 ± 2.116	28.273 ± 5.436	<0.001*
HCV RNA (IU/mL)	1114033.650 ± 1451732.743	Not detected	

ALT, alanine aminotranferase; AST, aspartate transaminase; BIL-T, total bilirubin; BIL-D, direct bilirubin; INR, international normalized ratio; PT, prothrombin time; Hb, hemoglobin; WBCs, white blood cells; GGT, gamma-glutamyl transferase; HDL, high-density lipoprotein; LDL, low-density lipoprotein and MDA, malondialdehyde.

*Values are presented as mean (SD). *Significant at *p* < 0.05.

Table 5 shows that there was a significant decrease in ALT, AST, total bilirubin, direct bilirubin, INR, prothrombin time, WBCs, GGT, and MDA (*p* < 0.001, *p* = 0.003, *p* = 0.004, *p* = 0.020, *p* = 0.006, *p* < 0.001, *p* = 0.014, *p* < 0.001, *p* < 0.001 respectively). However, there was a significant increase in albumin, platelets, cholesterol, LDL, HDL, and melatonin (*p* = 0.006, *p* < 0.001, *p* < 0.001, *p* < 0.001, *p* < 0.001, *p* < 0.001, *p* < 0.001 respectively). In addition, there was no significant change in either hemoglobin or serum creatinine levels (*p* = 0.507 and 0.724 respectively).

**TABLE 5:** Laboratories characterization of HCV-patients treated with triple therapy (Group III).

Parameter	Baseline	After 12 weeks of treatment	*p*-value
ALT, IU/L	91.685 ± 44.368	25.050 ± 3.137	<0.001*
AST, IU/L	53.235 ± 34.641	26.800 ± 3.302	0.003*
BIL-T, mg/dL	1.024 ± 0.204	0.894 ± 0.161	0.004*
BIL-D, mg/dL	0.202 ± 0.111	0.139 ± 0.011	0.020*
Albumin, g/dL	3.790 ± 0.484	4.115 ± 0.225	0.006*
INR, IU	1.200 ± 0.138	1.105 ± 0.081	<0.001*
PT, sec	14.612 ± 1.626	13.480 ± 0.574	<0.001*
Hb, g/dL	12.268 ± 1.562	12.499 ± 1.098	0.507
Platelets, 10^9^/L	129.050 ± 43.686	174.950 ± 38.465	<0.001*
WBCs, 10^9^/L	8.549 ± 2.140	7.203 ± 1.436	0.014*
Creatinine, mg/dL	0.958 ±0.196	0.938 ± 0.145	0.724
GGT, U/L	42.100 ± 6.512	32.750 ± 2.511	<0.001*
Cholesterol, mg/dL	161.950 ± 5.356	210.450 ± 10.782	<0.001*
LDL, mg/dL	114.835 ± 7.251	140.920 ± 13.539	<0.001*
HDL, mg/dL	41.000 ± 2.884	44.650 ± 3.117	<0.001*
MDA, nmol/mL	4.435 ± 0.292	2.555 ± 0.307	<0.001*
Melatonin, pg/mL	17.895 ± 1.927	43.890 ± 4.106	<0.001*
HCV RNA, IU/mL	955572.300 ± 4176787.175	Not detected	—

ALT, alanine aminotranferase; AST, aspartate transaminase; BIL-T, total bilirubin; BIL-D, direct bilirubin; INR, international normalized ratio; PT, prothrombin time; Hb, hemoglobin; WBCs, white blood cells; GGT, gamma-glutamyl transferase; HDL, high-density lipoprotein; LDL, low-density lipoprotein and MDA, malondialdehyde.

*Values are presented as mean (SD). * Significant at *p* < 0.05.

**FIGURE 2:**
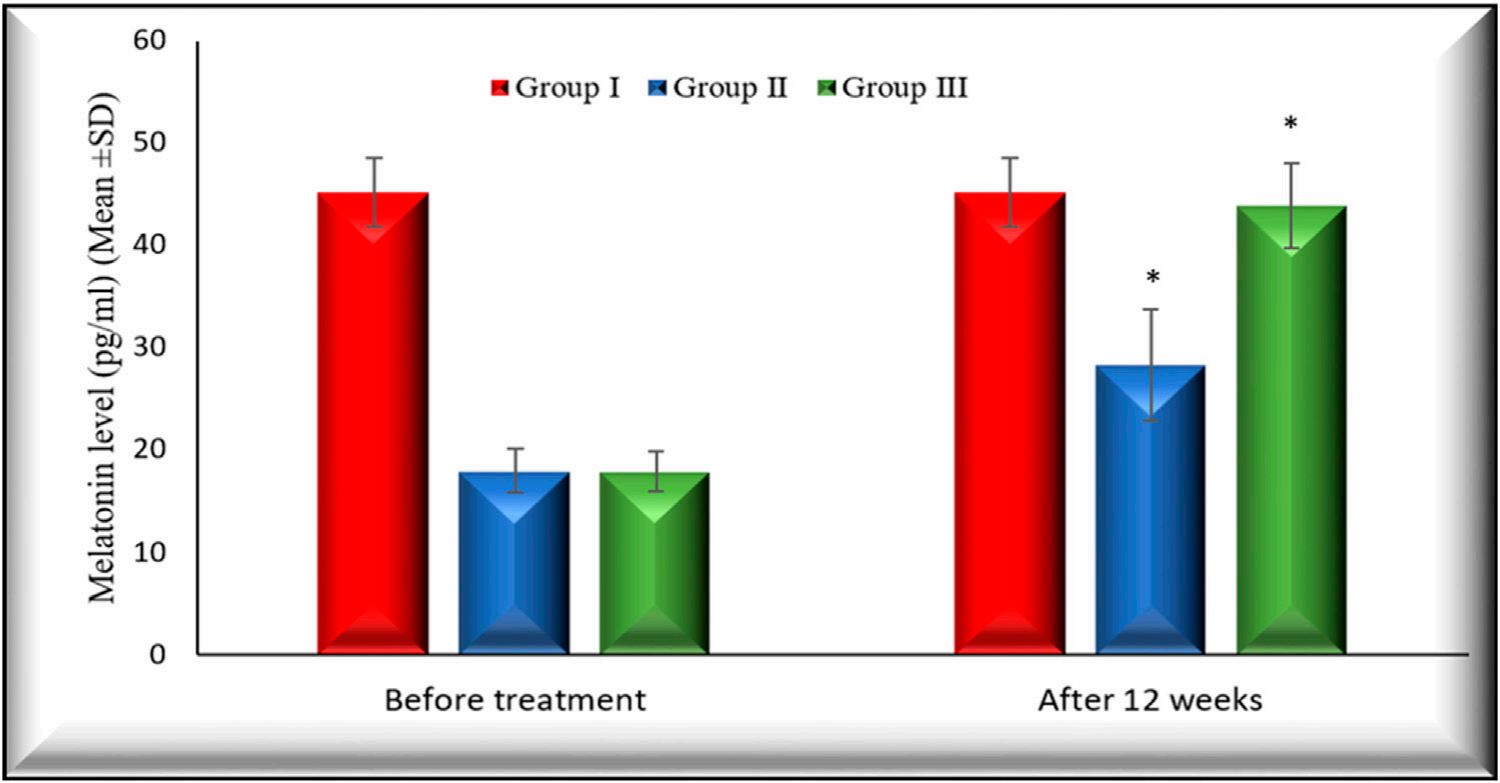
Changes in melatonin levels in the 3 studied groups before treatment and 3 months after treatment. Melatonin levels in both group II and group III increased significantly (*p* < 0.001) 3 months after treatment in comparison with their baseline. Group I: healthy control. Group II: CHC patients treated with sofosbuvir and daclatasvir. Group III: CHC patients treated with sofosbuvir, daclatasvir and ribavirin. Values are presented as mean (SD). *Significant difference.

**FIGURE 3:**
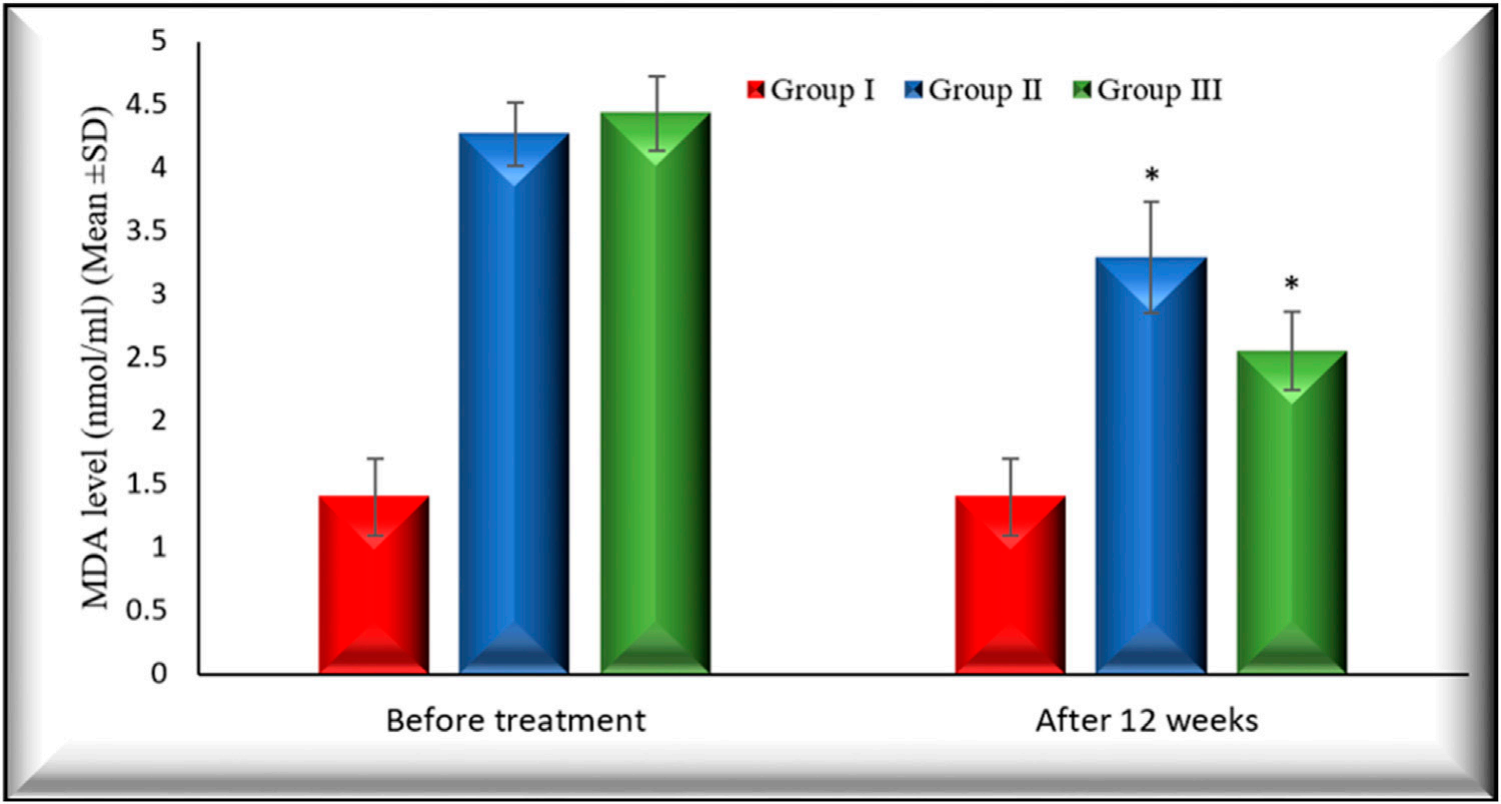
Changes in MDA level in the 3 studied groups before treatment and 3 months after treatment. MDA levels in both group II and group III decreased significantly (*p* < 0.001) 3 months after treatment in comparison with their baseline. Group I: healthy control. Group II: CHC patients treated with sofosbuvir and daclatasvir. Group III: CHC patients treated with sofosbuvir, daclatasvir, and ribavirin Values are presented as mean (SD). *Significant difference.

Figures 2, 3 illustrate the changes in melatonin and MDA in the 3 study groups before and 3 months after treatment.

The percentage of changes after 12 weeks of treatment on MDA in group II and group III is (22.47% (*p* < 0.001), 42.15(*p* < 0.001), respectively).

Also, the percentage of changes after 12 weeks of treatment on melatonin in group II and group III is (60.02% (*p* < 0.001), 146.11 (*p* < 0.001), respectively).

**FIGURE 4:**
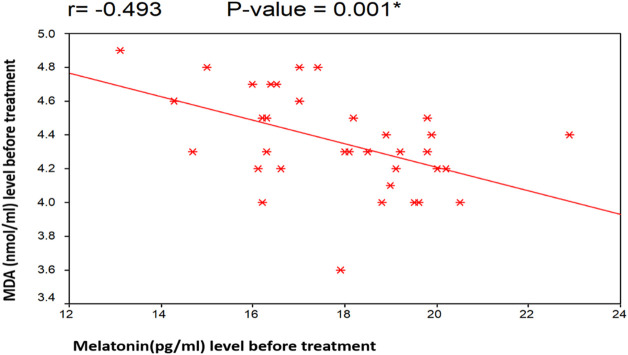
Illustrate the Pearson correlation analysis between the measured variables, which revealed the presence of a significant negative correlation between MDA and melatonin before treatment (*r* = −0.493 [*p* = 0.001]). Group II: CHC patients treated with sofosbuvir and daclatasvir. Group III: CHC patients treated with sofosbuvir, daclatasvir, and ribavirin.

**FIGURE 5:**
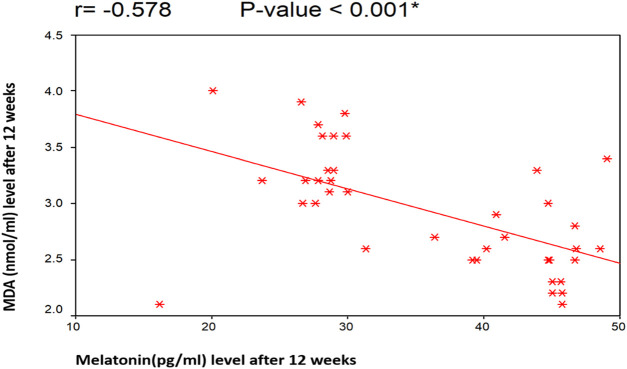
Illustrate the Pearson correlation analysis between the measured variables, which revealed the presence of a significant negative correlation between MDA and melatonin after treatment (*r* = −0.578 [*p* < 0.001]). Group II: CHC patients treated with sofosbuvir and daclatasvir. Group III: CHC patients treated with sofosbuvir, daclatasvir, and ribavirin.

Figures 4, 5 illustrate the Pearson correlation between melatonin and MDA in the 3 study groups before and 3 months after treatment

There was a negative non-significant correlation between melatonin and both total cholesterol and LDL before treatment (r= −0.049 [*p* = 0.766], r= −0.164 [*p* = 0.312], respectively). Also, there was a positive non-significant correlation between melatonin and HDL (r= 0.075 [*p* = 0.646]. In addition, MDA had a negative non-significant correlation with total cholesterol, LDL and HDL (r= −0.239 [*p* = 0.138], r= −0.091 [*p* = 0.577], r= −0.053 [*p* = 0.745], respectively).

Table 6 shows that Fatigue and headache were the most commonly reported adverse effects of DAAs.

**TABLE 6:** Side effects among studied groups.

Parameters	Group II (*n*= 20)	Group III (*n*= 20)
Anorexia	2 (10%)	3 (15%)
Decreased appetite	3 (15%)	4 (20%)
Itching	1 (5%)	0 (0%)
Headache	6 (30%)	7 (35%)
Fatigue	8 (40%)	10 (50%)
Fever	4 (20%)	5 (25%)
Serious adverse events or death	0 (0%)	0 (0%)

## Discussion

This study was dedicated to investigating the efficacy of using sofosbuvir/daclatasvir ± ribavirin on lipid peroxidation and melatonin levels in HCV patients. The results of this study suggest that sofosbuvir and daclatasvir, with or without ribavirin, were successful in decreasing lipid peroxidation and increasing melatonin levels. Infection with HCV affects millions of people worldwide. In Egypt, this infection’s prevalence rate is as high as 19% (Abdalla et al., 2005).

With the introduction of numerous novel antivirals, notably DAAs, the area of HCV treatment has undergone a significant transformation. Combinations of these medications in IFN-free regimens are now the norm for treating HCV infection due to their superior safety profiles and antiviral effectiveness.

Clinical study findings and preliminary results from real-world applications show that the combination of sofosbuvir, an NS5B inhibitor, and an NS5A inhibitor, such as the first-in-class drug daclatasvir, is one of the most effective antiviral medicines available. Sofosbuvir and an NS5A inhibitor given together for 12 weeks in combination with ribavirin seem to be a very good option for treating cirrhotic and treatment-experienced patients with any stage of fibrosis, regardless of the severity of the underlying liver disease and the baseline characteristics of the patients (Pol et al., 2016).

In the current study, basal serum MDA levels were shown to be significantly higher for CHC disease than for the comparable normal value. Other studies have reported similar outcomes (Romero et al., 1998; Seren et al., 2011). CHC is typically linked to an excess of oxidants and a deficiency of antioxidants. Free radicals produce lipid peroxidation, which results in the oxidative breakdown of polyunsaturated fatty acids that are essential to cellular membranes. When they are destroyed, harmful and reactive aldehyde metabolites like MDA are created (Levent et al., 2006).

Before treatment, the levels of serum MDA were significantly higher in patients in group II and group III than in the controls (*p* < 0.001). At the end of treatment, there was a significant decrease in serum MDA (*p* < 0.001). This agrees with Villani Rosanna et al. (Villani et al., 2020).

This data supports the idea that DAAs therapy partially reverses this negative effect by lowering the formation of reactive species caused by HCV. This may help partially explain the observed decrease in circulating MDA levels, compared to prior therapy in our patients.

A substantial association between the HCV virus and intracellular lipids has been observed in studies on the HCV life cycle, indicating that host lipids are crucial for viral replication (Vogt et al., 2013). Host blood lipid levels have an impact on the movement of hepatitis C virion and entry into hepatocytes. A fraction of host triacylglycerol-rich lipoproteins, also referred to as lipoviroparticles, complexes with circulating HCV particles (Schaefer and Chung, 2013).

Before treatment, the levels of serum LDL-C, high-density lipoprotein-cholesterol, and cholesterol were significantly lower in patients in group II and group III than the controls (*p* = 0.014, *p* < 0.001, *p* =0.004 respectively). At the end of treatment, there was a significant increase in serum LDL-C, high-density lipoprotein-cholesterol, cholesterol, and cholesterol (*p* = 0.026, *p* < 0.001, *p* < 0.001, respectively), This is in agreement with Ayman M. El-Lehleh et al. (El-Lehleh et al., 2019).

This effect is attributed to a reversal of the impact of HCV replication on hepatic lipid metabolism.

In the current study, we observed that HCV patients produced less endogenous melatonin. This is in agreement with Vikram Mehraj et al. (Mehraj and Routy, 2015). The accumulation of toxic chemicals caused by hepatic insufficiency or pineal dysfunction may be the mechanism for decreased melatonin production in people with liver illness. Another theory is that persistent viral infections manipulate the host immune system to develop illness tolerance through kynurenine catabolites. HCV infection indoleamine-2, 3-dioxygenase (IDO) expression was reported by (Lepiller et al., 2015). These study findings showed that HCV infection directly induced IDO, which could lead to decreased melatonin levels.

Group III has a higher level of melatonin after treatment compared with group II. This enhanced efficacy of ribavirin combination therapy in this group may be the result of ribavirin’s ability to induce a cellular immune reaction against HCV. Ribavirin acts on Human peripheral blood mononuclear cells (PBMCs) and induces T helper (Th) differentiation. T cells have the four enzymes required for the synthesis of melatonin (aromatic L-amino acid decarboxylase, arylalkylamine N-acetyltransferase, N- acetylserotonin methyltransferase, and tryptophan hydroxylase) and produce high levels of melatonin (Shiina et al., 2004; Ren et al., 2017).

Despite not being an antioxidant, ribavirin’s antiviral properties might reduce viral load and inflammation. This process could additionally reduce oxidative stress caused on by viruses and MDA in group III compared to group II (Levent et al., 2006).

In the current study, we found that DAAs have a safe profile, as no patient in our series stopped treatment due to severe adverse events, which is in line with (Abd El Rhman. et al., 2019).

Based on our study results, HCV decreases melatonin level, which enables it to evade the immune system and develop illness tolerance. We therefore urge the conduct of further clinical studies to examine the advantages of melatonin supplements as an adjunctive therapy to antiviral medication in hepatitis C patients. Also, our result shows that after using DAA, we found a significant increase in melatonin levels compared to before treatment. Consequently, other studies are needed to explain the mechanism by which DAAs increase melatonin production. In addition, our data showed that there was a significant negative correlation between serum MDA and serum melatonin concentrations in CHC patients.

From the present study, it is established that CHC is associated with oxidative stress, as evidenced by an increased MDA level and a decrease in melatonin level. Further study is needed with a large sample size.

## Conclusion

In conclusion, these preliminary findings suggest that DAAs increase endogenous melatonin and decrease MDA levels, which are associated with improved antiviral treatment outcomes in patients with HCV.

## Data Availability

The raw data supporting the conclusion of this article will be made available by the authors, without undue reservation.

## Abbreviations

CHC, chronic hepatitis C; DAAs, direct-acting antivirals; HCV, hepatitis C virus; HCC, hepatocellular carcinoma; IFN, Interferon; MDA, malondialdehyde; ROS, reactive oxygen species.
